# Supplementary material for: Pre-configuring chromatin architecture with histone modifications guides hematopoietic stem cell formation in mouse embryos
Source: Nat Commun. 2022 Jan 17;13:346. doi: 10.1038/s41467-022-28018-z (PMC8764075; doi:10.1038/s41467-022-28018-z)
Supplement: Supplementary file 1 — Supplementary Information [file 41467_2022_28018_MOESM1_ESM.pdf]

Supplementary Information

Pre-configuring chromatin architecture with histone modifications guides hematopoietic stem cell formation in mouse embryos

Chen C. Li[1,5], Guangyu Zhang[2,5], Junjie Du[2], Di Liu[1], Zongcheng Li[3], Yanli Ni[3], Jie Zhou[3], Yunqiao Li[2], Siyuan Hou[4], Xiaona Zheng[2], Yu Lan[4,*], Bing Liu[2,3,4,*] & Aibin He[1,*]

[1]*Beijing Key Laboratory of Cardiometabolic Molecular Medicine, Institute of Molecular Medicine, Peking-Tsinghua Center for Life Sciences, Peking University, Beijing 100871, China*

[2]*State Key Laboratory of Proteomics, Academy of Military Medical Sciences, Academy of Military Sciences, Beijing, 100850, China*

[3]*State Key Laboratory of Experimental Hematology, Institute of Hematology, Fifth Medical Center of Chinese PLA General Hospital, Beijing, 100850, China.*

[4]*Key Laboratory for Regenerative Medicine of Ministry of Education, Institute of Hematology, School of Medicine, Jinan University, Guangzhou, China.*

[5]*These authors contributed equally to this work*

***Correspondence**: Aibin He (ahe@pku.edu.cn), Bing Liu (bingliu17@yahoo.com) and Yu Lan (rainyblue_1999@126.com)*

This file contains 7 supplementary figures, figure legends and 3 supplementary tables.

Supplementary Fig. 1

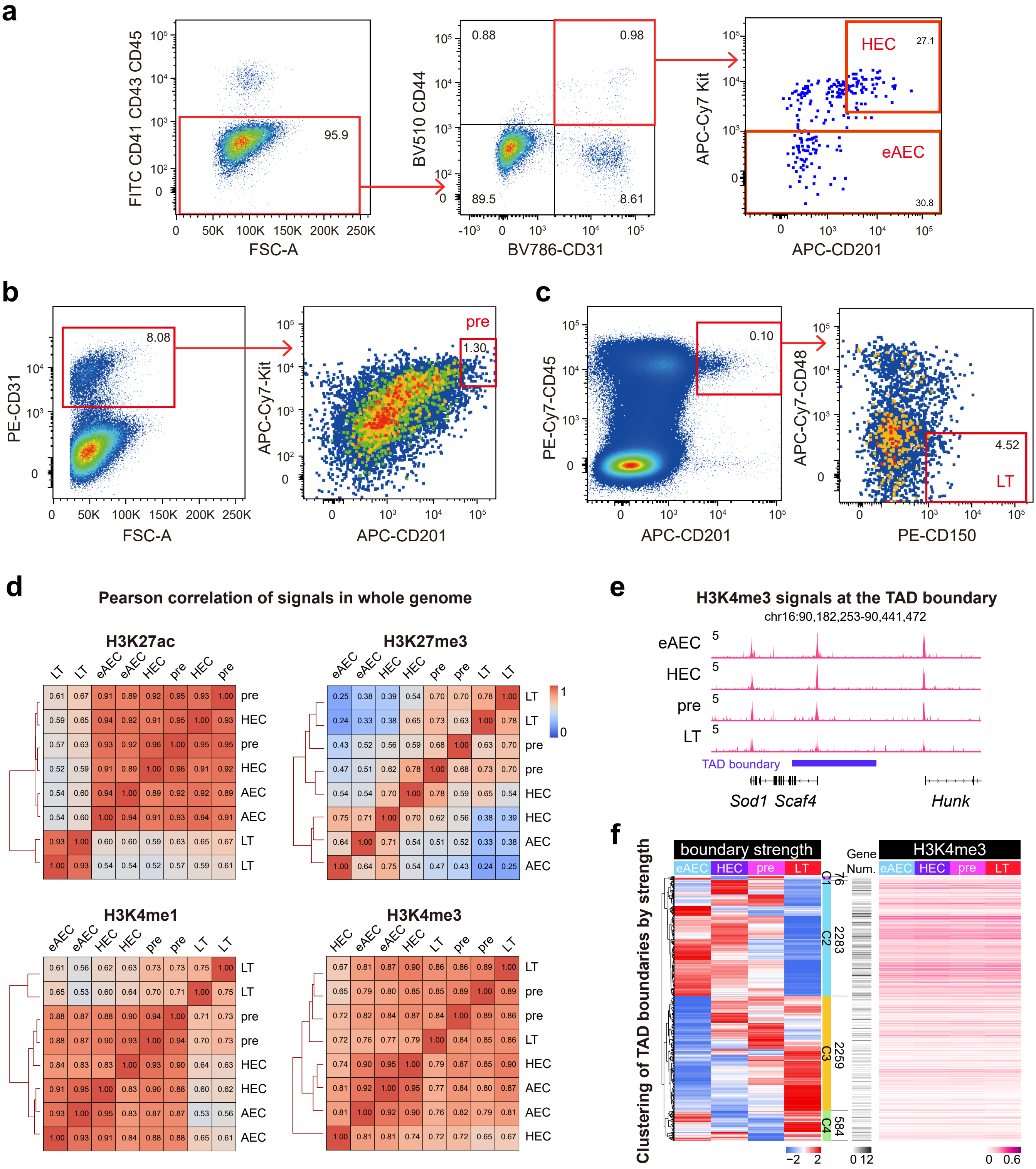

Supplementary Fig. 1: FACS strategies. a Surface markers and gating strategies in FACS of eAECs and HECs. **b** Surface markers and gating strategies in FACS of pre-HSCs (pre). **c** Surface markers and gating strategies in FACS of LT-HSCs (LT). **d** Pearson correlation analysis with replicates of H3K27ac, H3K27me3, H3K4me1 and H3K4me3 itChIP-seq experiments genome wide. Non-duplicated reads of all replicates passing filter were normalized to the same level for further analysis. **e** Track view showing an example of TAD boundary enriched for H3K4me3. **f** Hierarchical clustering of all TAD boundaries by boundary strengths with z-scaled rows. TAD boundaries were merged from boundaries identified in four cell populations. The corresponding gene numbers and H3K4me3 signals at TAD boundary regions (40 kb) were shown on the right. eAEC, early AEC; pre, pre-HSC; LT, LT-HSC.

Supplementary Fig. 2

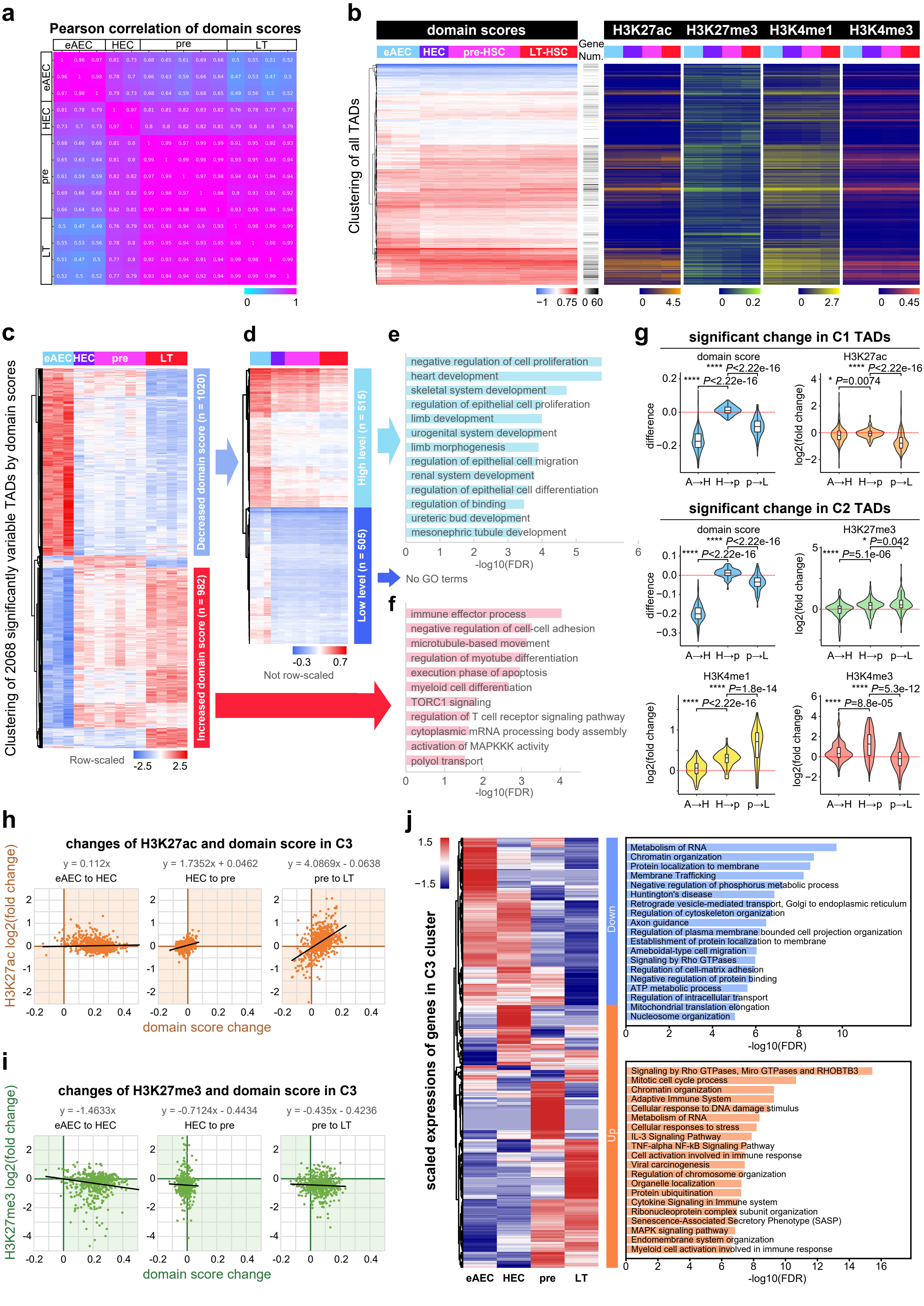

Supplementary Fig. 2: Dynamic features of the intra-TAD connectivity and multiple histone modifications. a Pearson correlation analysis with TAD domain scores calculated from biological replicates of Hi-C data. After normalization and correction, Hi-C matrices at 40 kb bins were used for calculation of TAD domain scores. **b** Hierarchical clustering of all TADs by normalized domain scores, alongside histone modifications in ChIP-seq peaks within TADs. **c** Hierarchical clustering of the 2,068 TADs with the cutoff FDR < 0.001 by normalized domain scores. The clustering was performed with row-z-scaled values. **d** Further Hierarchical clustering of 1,020 TADs with decreased domain scores by normalized domain scores (no row scaled). **e** Biological processes in GO analysis of 515 TADs with decreased but high domain scores. **f** Biological processes in GO analysis of 982 TADs with increased domain scores. The GO analysis was done by GREAT. *P*-value was calculated by two-sided binomial test and adjusted by BH correction (FDR). **g** Violin plots showing changes of domain scores and significantly variable histone modifications in C1 TADs (n = 200) and C2 TADs (n = 148). Box-and-whiskers plots represented the maxima, 75th percentile, median, 25th percentile, and minima. Wilcoxon Rank Sum test (two-side) was performed between two adjacent stages. *, *P*-value <0.05; **, *P*-value<0.01; ***, *P*-value<0.001; ****, *P*-value<0.0001; "ns", not significant. **h,i** Scatter plots showing the normalized change trend of TAD domain score and H3K27ac signals (**h**) or H3K27me3 signals (**i**) of TADs in C3 cluster with increasing domain scores. Each dot represented a TAD. *Y*-axis: Log2(fold change) of histone modifications in HEC/AEC, pre/HEC, and LT/pre; *X*-axis: The difference of TAD domain score between adjacent two populations. **j** Heatmap showing sub-clustering by gene expression in C3 cluster TADs, with the increasing domain scores. The GO analysis was done by Metascape. *P*-value was calculated by two-side hypergeometric test and adjusted by BH correction (FDR). eAEC, early AEC; pre, pre-HSC; LT, LT-HSC.

Supplementary Fig. 3

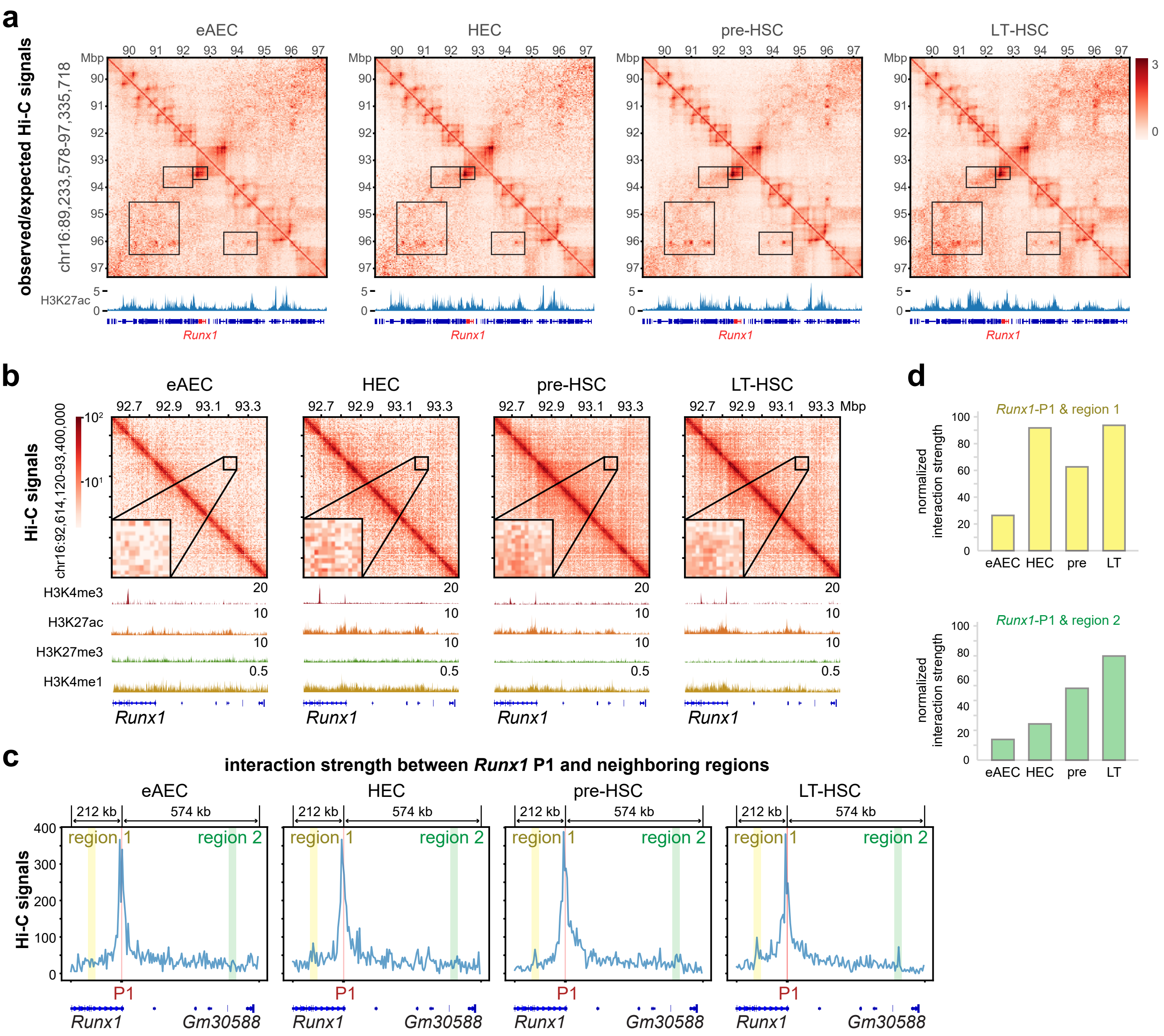

Supplementary Fig. 3: Examples of changing intra-TAD connectivity and multiple histone modifications. **a** Contact heatmap exemplifying short-range contacts and long-range contacts around *Runx1* gene within TADs, enhanced from eAEC through HEC and pre-HSC to LT-HSC. The boxs indicate examples of specific interactions increasing or appearing from eAECs to LT-HSCs. Visualization was performed with observed/expected Hi-C matrices at 5 kb resolution from a large view. **b** Exemplification showing the increasing intra-TAD interactions associated with feature histone modifications at *Runx1* gene site. This visualization was performed with depth-normalized Hi-C matrices (5 kb resolution) with observed treatment. **c** Quantification curves for the changing loop strength between the *Runx1* P1 promoter and neighboring regions. The quantification was performed from normalized Hi-C matrices (5 kb resolution) with observed signals. The *Runx1* P1 region in red was TSS ± 2.5 kb. The region 1 in yellow overlapped with *Runx1* P2 promoter, while the region 2 in green represented a distal enhancer. **d** Bar plot showing the relative interaction strengths between *Runx1* P1 and two regions as indicated in yellow or green in (**c**). eAEC, early AEC; pre, pre-HSC; LT, LT-HSC.

Supplementary Fig. 4

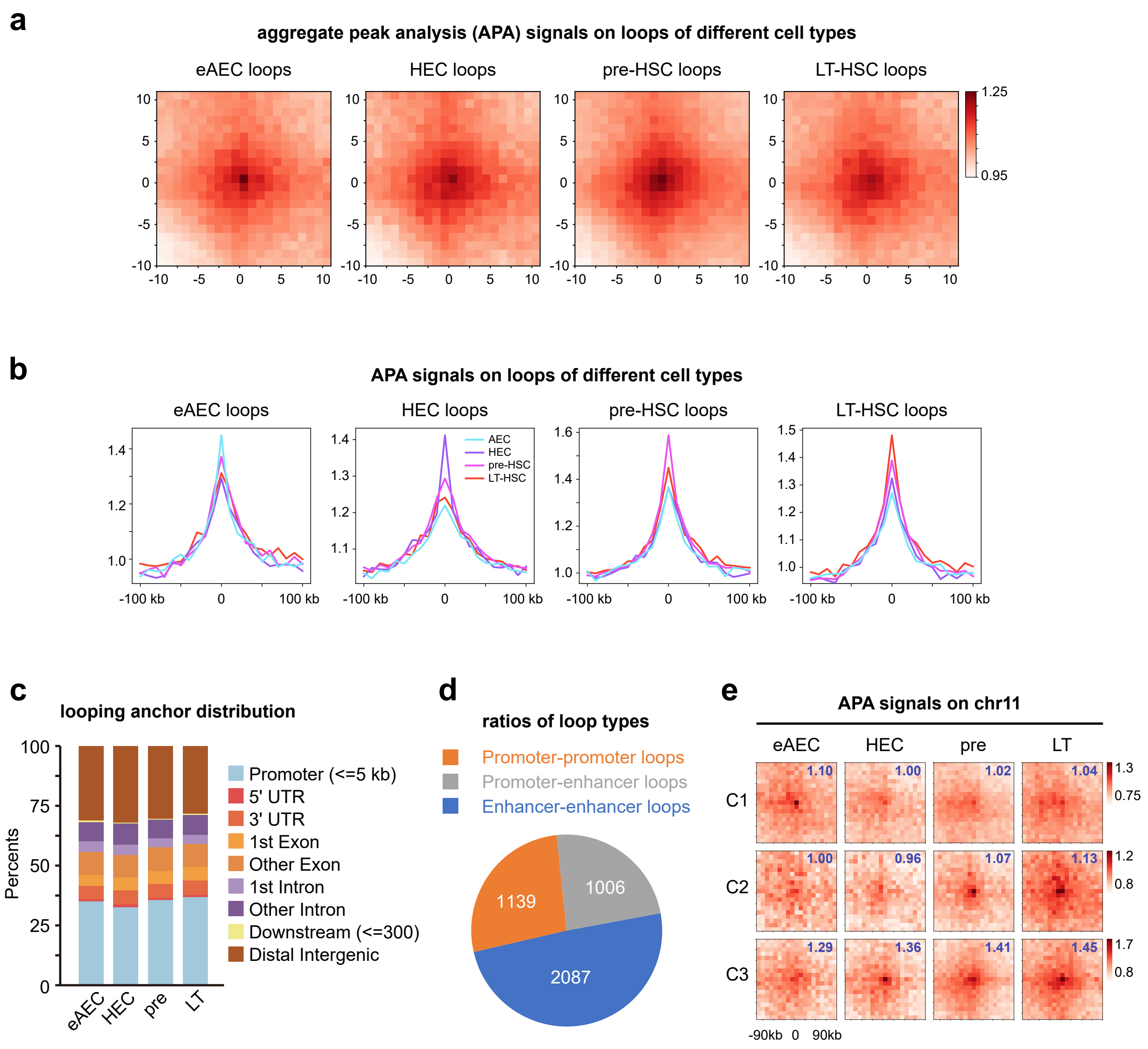

Supplementary Fig. 4: Changing loop structures during LT-HSC formation. a Heatmaps showing the aggregate peak analysis (APA) signals on loops indentified in each cell population. The Hi-C matrices at 10 kb resolution were used for loop calling. **b** Curves quantifying the APA signals on loops indentified in each cell population, with Hi-C signals of four cell populations. The Hi-C matrices at 10 kb resolution were used for loop calling. **c** Bar plot showing the distribution of looping anchor regions. Loops were identified with matrices at 10 kb bins in four cell populations. **d** Pie chart showing the portion of different types of loops involving promoters (TSS ± 5 kb) and enhancers (defined by distal H3K27ac ChIP-seq peaks). **e** APA heatmap showing the changing loop strengths in three types of loops among enhancers and promoters. Each pixel was 10 kb x 10 kb. In x or y axis, the region shown was from -90 kb (-9 pixels) to + 90 kb (+9 pixels) around the center 10-kb pixel. The values shown were the average APA scores of the centric 30 kb x 30 kb regions. Chr11 was chosen due to the largest number of top 1,000 variable loops identified above.

Supplementary Fig. 5

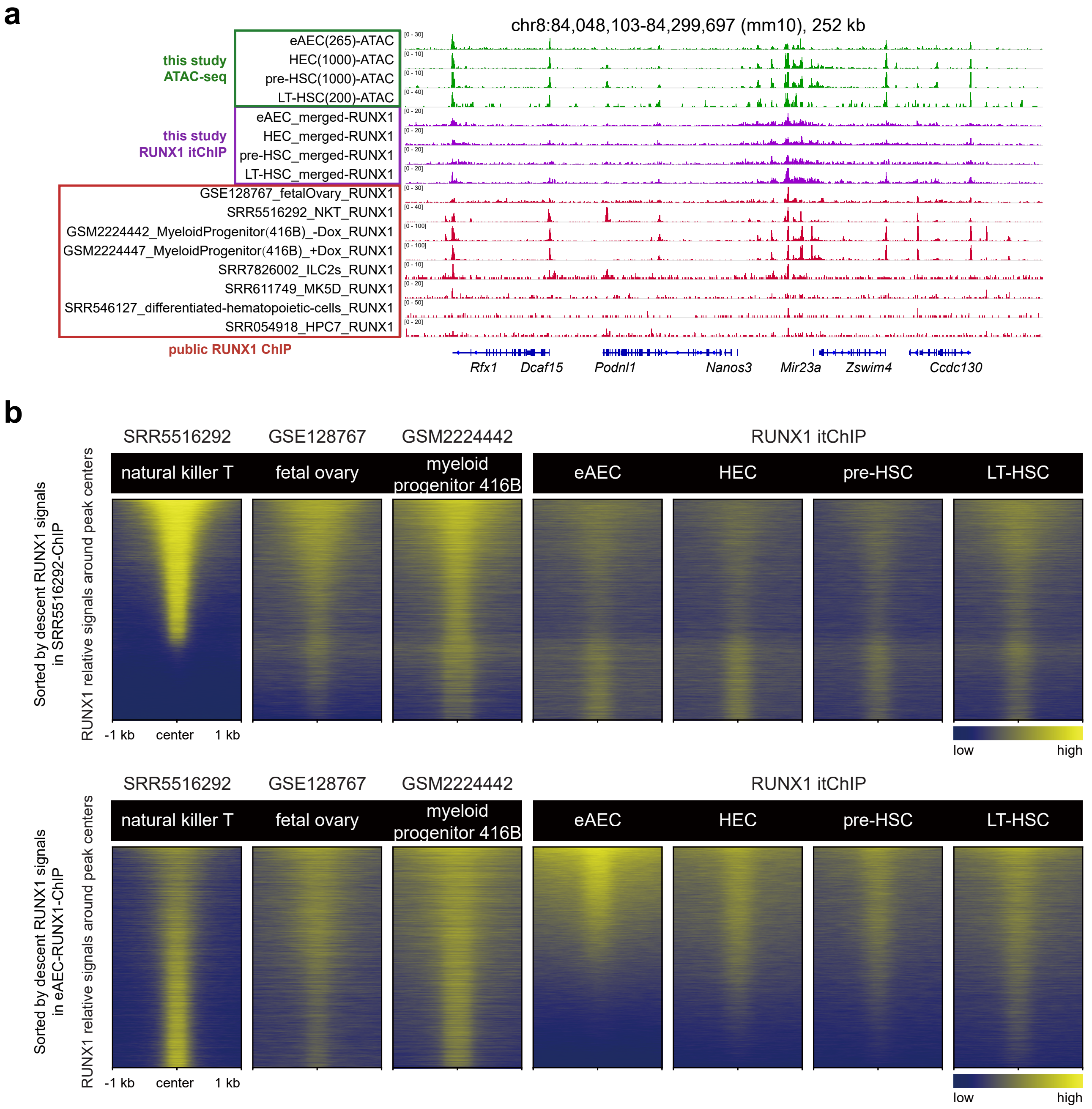

Supplementary Fig. 5: Comparison of RUNX1 itChIP-seq data with public RUNX1 ChIP-seq data of other cell types. a Track view showing the visualization comparison of our RUNX1 itChIP-seq data with public RUNX1 ChIP-seq data from other cell types. There were common peaks sharing the same binding motif, as well as unique peaks reflecting the cell type specificity. **b** Heatmaps showing the cell type specific enrichment signals of RUNX1 binding in our data and public data. The regions were peaks merged from all seven RUNX1 ChIP-seq data sets. Regions in the upper part heatmaps were sorted by descent RUNX1 signals around peak-center ±1 kb in SRR5516292-ChIP. Regions in the lower part heatmaps were sorted by descent RUNX1 signals around peak-center ±1 kb regions in eAEC RUNX1-itChIP. eAEC, early AEC; pre, pre-HSC; LT, LT-HSC.

Supplementary Fig. 6

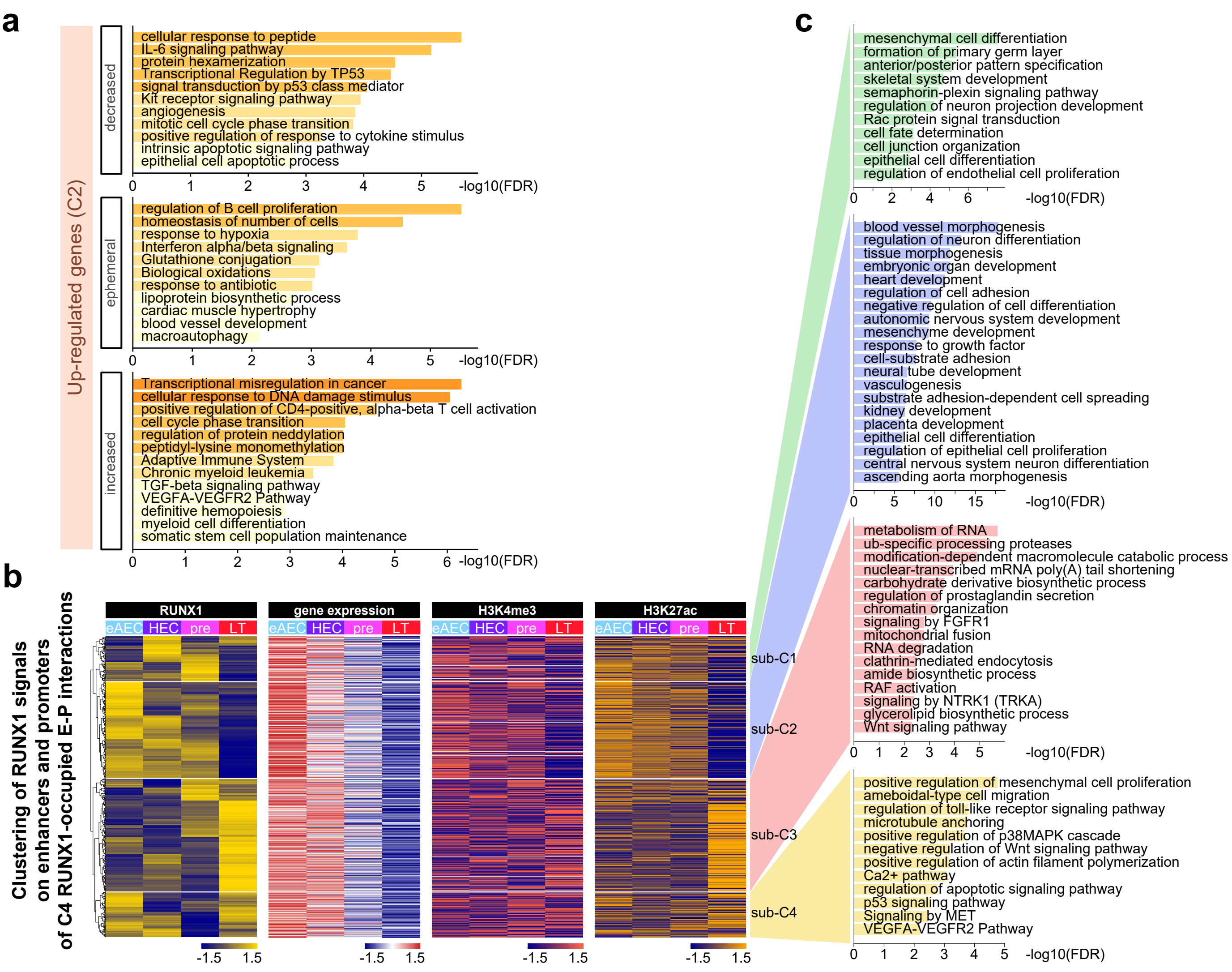

Supplementary Fig. 6: Sub-clustering analysis of C2 and C4 RUNX1-engaged E-P interactions. a Biological processes in Gene Ontology analysis with genes in three sub-clusters of C2 RUNX1-engaged E-P interactions in Fig. 4f. The GO analysis was done by Metascape. *P*-value was calculated by two-side hypergeometric test and adjusted by BH correction (FDR). **b** Clustering of genes in C4 as in Fig. 4c by RUNX1 binding intensity (row z-scaled) at enhancer and promoter loops. Corresponding gene expression, H3K27ac and H3K4me3 signals were shown on the right. The ANOVA *P*-values (one-side) of expression or signals in sub-C2 cluster were 1.07e-10 for RUNX1, <2e-16 for gene expression, 6.53e-09 for H3K4me3 and <2e-16 for H3K27ac. The ANOVA *P*-values of expression or signals in sub-C3 cluster were 3.99e-09 for RUNX1, <2e-16 for gene expression, 0.275 for H3K4me3 and 2.31e-12 for H3K27ac. **c** Enrichment of biological processes by Gene Ontology analysis of four clusters in (**b**) by metascape were shown. *P*-value was calculated by two-side hypergeometric test and adjusted by BH correction (FDR). eAEC, early AEC; pre, pre-HSC; LT, LT-HSC.

Supplementary Fig. 7

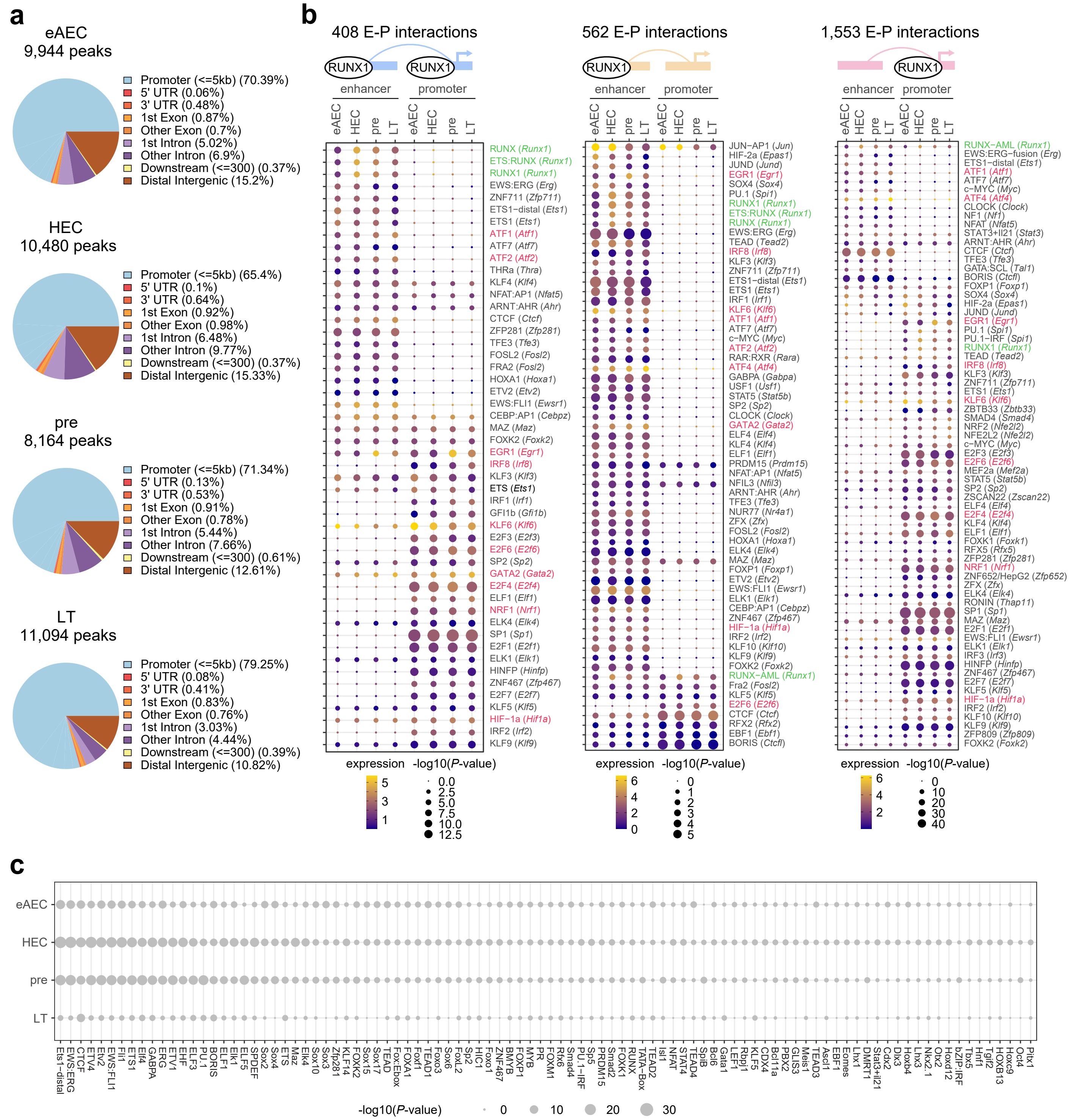

Supplementary Fig. 7: Putative RUNX1 co-factors involved in enhancer-promoter interactions. a Pie chart showing genomic annotation of all RUNX1 peaks. **b** Bubble plot showing TF candidates binding on enhancers or promoters from three types of RUNX1-engaged E-P interactions. The color indicates gene expression and the size of circles is proportional to the significance of TF motif enrichment. Gene expression was calculated by log2(TPM/10+1). Genes in brackets were used for gene expression calculation. The significance of TF motifs was calculated by -log10(*P*-value). TF motifs were identified by Homer. RUNX1-related motifs were marked in green. TF motifs in red were related with hematopoiesis and immune. **c** Bubble plot showing TF motifs found in enhancers and promoters of 3,463 E-P interactions without RUNX1 occupancy as in Fig. 4b. This set served as acontrol for TF motif identification from RUNX1-engaged E-P interactions. TF motif analysis in (**b**) or (**c**) was performed by HOMER. *P*-value was calculated by two-sided binomial test. eAEC, early AEC; pre, pre-HSC; LT, LT-HSC.

Supplementary Table 1. Data quality control of Hi-C libraries

Stage	Replicate	Final reps	Cell number	Total Read pairs	Mapping pair rate	Valid Read pairs	Nonduplicated Read pairs	Cis (>10kb)	Trans
AEC	AEC-hic-rep1_part1	AEC-rep1	1000	439.1 M	50.8%	188.6 M	85.1 M	56.5 M	18.9 M
	AEC-hic-rep1_part2		1000	294.8 M	59.1%	160.0 M	58.9 M	33.6 M	20.5 M
	AEC-hic-rep2_part1	AEC-rep2	1000	451.1 M	51.5%	207.0 M	80.0 M	50.0 M	21.1 M
	AEC-hic-rep2_part2		1000	506.8 M	55.0%	238.1 M	83.7 M	55.5 M	20.1 M
	AEC-hic-rep3_part1	AEC-rep3	1000	284.7 M	47.2%	100.5 M	32.8 M	19.1 M	10.6 M
	AEC-hic-rep3_part2		1000	512.9 M	60.2%	288.4 M	122.8 M	71.6 M	41.0 M
HEC	HEC-hic-rep1_part1	HEC-rep1	286	433.4 M	52.9%	211.1 M	48.17 M	35.1 M	7.90 M
	HEC-hic-rep1_part2		539	453.1 M	52.0%	207.7 M	72.30 M	52.9 M	9.30 M
	HEC-hic-rep2	HEC-rep2	911	517.2 M	59.7%	283.5 M	125.5 M	88.2 M	22.4 M
pre-HSC	preHSC-hic-rep1	preHSC-rep1	1068	696.0 M	67.3%	438.2 M	168.7 M	120.3 M	24.4 M
	preHSC-hic-rep2	preHSC-rep2	1083	664.8 M	64.6%	329.0 M	137.4 M	94.0 M	24.6 M
	preHSC-hic-rep3	preHSC-rep3	1100	571.2 M	64.4%	341.2 M	154.8 M	109.4 M	26.2 M
	preHSC-hic-rep4	preHSC-rep4	1368	470.0 M	63.8%	272.4 M	122.5 M	89.1 M	18.4 M
	preHSC-hic-rep5	preHSC-rep5	963	608.7 M	65.3%	363.3 M	169.3 M	120 M	26.0 M
LT-HSC	LTHSC-hic-rep1	LTHSC-rep1	576	618.0 M	61.7%	331.6 M	99.0 M	64.1 M	22.3 M
	LTHSC-hic-rep2	LTHSC-rep2	636	552.9 M	59.9%	285.4 M	90.0 M	63.6 M	13.1 M
	LTHSC-hic-rep3	LTHSC-rep3	637	590.0 M	63.1%	340.0 M	93.3 M	65.0 M	16.1 M
	LTHSC-hic-rep4	LTHSC-rep4	832	527.8 M	63.3%	306.5 M	92.1 M	65.8 M	16.6 M

Supplementary Table 2. Data quality control of histone modification itChIP-seq libraries

	Sample	Cell number	Raw fragments	Filtered fragments	Mapping rates	Unique mapped reads	Non-duplicated reads	Duplication-rates
AEC	H3K27ac_rep1	500	10,510,463	10,507,784	98.50%	18,599,738	6,763,704	63.64%
	H3K27ac_rep2	500	13,789,768	13,784,905	97.98%	23,783,096	4,396,107	81.52%
	H3K27me3_rep1	500	5,906,635	5,897,582	97.65%	8,260,312	3,886,443	52.95%
	H3K27me3_rep2	500	16,156,491	16,150,101	97.33%	24,536,043	3,494,499	85.76%
	H3K4me1_rep1	500	12,521,313	12,518,174	98.40%	21,379,017	15,010,232	29.79%
	H3K4me1_rep2	500	16,652,177	16,644,846	97.74%	27,672,090	14,276,231	48.41%
	H3K4me3_rep1	500	12,013,929	12,009,108	97.20%	16,740,476	1,673,356	90.00%
	H3K4me3_rep2	500	13,947,703	13,941,052	97.43%	22,367,433	693,959	96.90%
HEC	H3K27ac_rep1	525	15,472,783	15,467,357	98.54%	28,185,348	13,580,555	51.82%
	H3K27ac_rep2	500	10,787,549	10,784,744	98.23%	18,510,945	12,780,754	30.96%
	H3K27me3_rep1	525	14,801,100	14,796,667	97.51%	22,930,441	9,629,703	58.00%
	H3K27me3_rep2	500	14,969,111	14,964,112	97.70%	22,218,467	11,739,333	47.16%
	H3K4me1_rep1	525	19,889,426	19,882,937	98.36%	34,638,087	22,467,488	35.14%
	H3K4me1_rep2	500	23,589,286	23,578,466	97.56%	39,347,541	31,554,216	19.81%
	H3K4me3_rep1	525	14,607,920	14,602,133	97.08%	20,263,231	1,578,457	92.21%
	H3K4me3_rep2	500	16,076,841	16,070,469	97.61%	25,536,104	7,100,878	72.19%
pre-HSC	H3K27ac_rep1	504	17,155,031	17,148,318	98.45%	31,050,661	16,136,014	48.03%
	H3K27ac_rep2	461	13,106,503	13,102,869	98.25%	22,824,339	6,986,820	69.39%
	H3K27me3_rep1	504	15,823,634	15,818,403	97.53%	23,130,282	11,010,234	52.40%
	H3K27me3_rep2	461	13,171,977	13,168,817	97.09%	17,893,224	3,985,740	77.72%
	H3K4me1_rep1	504	17,311,204	17,304,964	98.03%	29,341,072	20,959,418	28.57%
	H3K4me1_rep2	461	15,992,984	15,988,293	98.19%	26,953,190	18,487,808	31.41%
	H3K4me3_rep1	504	17,604,664	17,598,483	97.82%	27,502,374	5,680,359	79.35%
	H3K4me3_rep2	461	14,275,106	14,271,083	97.25%	20,078,034	4,340,274	78.38%
LT-HSC	H3K27ac_rep1	460	24,114,683	24,099,131	98.66%	43,487,467	11,165,839	74.32%
	H3K27ac_rep2	400	9,060,511	9,056,810	98.34%	15,672,147	4,879,064	68.87%
	H3K27ac_rep3	400	9,797,089	9,793,351	98.24%	16,690,734	10,238,064	38.66%
	H3K27me3_rep1	460	19,393,796	19,382,161	97.83%	27,910,276	5,105,836	81.71%
	H3K27me3_rep2	342	16,817,090	16,812,401	96.98%	21,008,394	4,883,936	76.75%
	H3K4me1_rep1	460	20,596,277	20,583,553	98.38%	34,194,258	17,854,736	47.78%
	H3K4me1_rep2	342	16,165,434	16,160,758	97.65%	24,772,992	16,579,536	33.07%
	H3K4me3_rep1	460	18,531,579	18,518,924	97.14%	23,728,743	855,185	96.40%
	H3K4me3_rep2	450	14,466,212	14,457,096	97.28%	20,761,147	1,198,801	94.23%
	H3K4me3_rep3	450	10,399,630	10,391,755	96.61%	13,740,117	1,295,093	90.57%

Supplementary Table 3. Data quality control of RUNX1 itChIP-seq libraries

	Sample	Cell number	Raw fragments	Filtered fragments	Mapping rates	Unique mapped reads	Non-duplicated reads	Duplication-rates
AEC	RUNX1_rep1	1000	18,542,102	18,522,530	92.21%	30,565,983	3,865,855	87.35%
	RUNX1_rep2	1000	21,174,095	21,152,774	92.56%	34,893,012	9,083,642	73.97%
	RUNX1_rep3	1119	10,556,127	10,551,434	97.96%	17,338,287	9,952,956	42.60%
HEC	RUNX1_rep1	790	13,017,786	13,012,608	97.75%	22,306,073	15,518,981	30.43%
	RUNX1_rep2	756	10,740,510	10,735,916	98.18%	18,157,202	10,238,967	43.61%
pre-HSC	RUNX1_rep1	1400	13,236,861	13,232,086	97.60%	22,256,590	17,615,506	20.85%
	RUNX1_rep2	1010	11,251,998	11,247,429	97.75%	17,991,640	8,740,291	51.42%
LT-HSC	RUNX1_rep1	1400	16,263,302	16,254,945	97.66%	25,477,630	16,023,496	37.11%
	RUNX1_rep2	1800	12,615,867	12,611,544	97.83%	21,024,286	7,159,948	65.94%
