# Supplementary material for: Pre-configuring chromatin architecture with histone modifications guides hematopoietic stem cell formation in mouse embryos
Source: Nat Commun. 2022 Jan 17;13:346. doi: 10.1038/s41467-022-28018-z (PMC8764075; doi:10.1038/s41467-022-28018-z)
Supplement: Supplementary file 3 — Reporting Summary [file 41467_2022_28018_MOESM3_ESM.pdf]

Corresponding author(s): Aibin He

Last updated by author(s): Dec 14, 2021

Reporting Summary

Nature Research wishes to improve the reproducibility of the work that we publish. This form provides structure for consistency and transparency in reporting. For further information on Nature Research policies, see our Editorial Policies and the Editorial Policy Checklist.

Statistics

For all statistical analyses, confirm that the following items are present in the figure legend, table legend, main text, or Methods section.

n/a	Confirmed	
☐	☒	The exact sample size (*n*) for each experimental group/condition, given as a discrete number and unit of measurement
☐	☒	A statement on whether measurements were taken from distinct samples or whether the same sample was measured repeatedly
☐	☒	The statistical test(s) used AND whether they are one- or two-sided<br>*Only common tests should be described solely by name; describe more complex techniques in the Methods section.*
☐	☒	A description of all covariates tested
☐	☒	A description of any assumptions or corrections, such as tests of normality and adjustment for multiple comparisons
☐	☒	A full description of the statistical parameters including central tendency (e.g. means) or other basic estimates (e.g. regression coefficient) AND variation (e.g. standard deviation) or associated estimates of uncertainty (e.g. confidence intervals)
☐	☒	For null hypothesis testing, the test statistic (e.g. *F*, *t*, *r*) with confidence intervals, effect sizes, degrees of freedom and *P* value noted<br>*Give P values as exact values whenever suitable.*
☐	☒	For Bayesian analysis, information on the choice of priors and Markov chain Monte Carlo settings
☐	☒	For hierarchical and complex designs, identification of the appropriate level for tests and full reporting of outcomes
☐	☒	Estimates of effect sizes (e.g. Cohen's *d*, Pearson's *r*), indicating how they were calculated

Our web collection on statistics for biologists contains articles on many of the points above.

Software and code

Policy information about availability of computer code

Data collection	All software used in this study has been described in published literature. There are details in the online Methods. Software version: FastQC (v 0.11.5), Cutadapt (v 1.11), Bowtie2 (v 2.2.9), Deeptools (v 2.2.3). Samtools (v 1.9), Picard (v.2.2.4), MACS2 (v.2.2.5), HiCExplorer (v.3.4.2), Cooler (v.0.8.5), PyGenomeTracks (v.1.0). Further data analysis and presentation are performed with R (v3.5.1) .
Data analysis	There are details in the online Methods. All parameters used to analyze data are described in methods section and custom scripts are available upon request. Cutadapt (v 1.11) is used for trimming and filtering with raw read inputs. Bowtie2 (v 2.2.9) is used for mapping. Samtools (v 1.9) is used for sorting and selecting uniquely mapping reads. Picard (v.2.2.4) is used for deduplication. Deeptools bamCoverage (v.3.3.1) is used to generate bigwig files. Deeptools (v 2.2.3) plotHeatmap is used for calculation and visualization of signals in interested regions. MACS2 (v.2.2.5) is used for peak calling of enriched signals. HiCExplorer (v.3.4.2) and Cooler (v.0.8.5) are applied for Hi-C data processing and calculation in levels of compartments, TADs and loops. PyGenomeTracks (v.1.0) performs combined visualization of Hi-C and ChIP-seq data. R (v3.5.1) is for statistical analysis and visualization.

For manuscripts utilizing custom algorithms or software that are central to the research but not yet described in published literature, software must be made available to editors and reviewers. We strongly encourage code deposition in a community repository (e.g. GitHub). See the Nature Research guidelines for submitting code & software for further information.

Data

Policy information about availability of data

All manuscripts must include a data availability statement. This statement should provide the following information, where applicable:

- Accession codes, unique identifiers, or web links for publicly available datasets
- A list of figures that have associated raw data
- A description of any restrictions on data availability

All sequencing data have been deposited at the NCBI Gene Expression Omnibus (GEO) with the accession number GSE161328.

Field-specific reporting

Please select the one below that is the best fit for your research. If you are not sure, read the appropriate sections before making your selection.

- [x] Life sciences
- [ ] Behavioural & social sciences
- [ ] Ecological, evolutionary & environmental sciences

For a reference copy of the document with all sections, see nature.com/documents/nr-reporting-summary-flat.pdf

Life sciences study design

All studies must disclose on these points even when the disclosure is negative.

Sample size	Two to five biological replicates for Hi-C or ChIP-seq libraries are sufficient for assessing the data quality.
Data exclusions	No data were excluded from the analysis.
Replication	As for ChIP-seq libraries, real-time qPCR is used for checking enrichment of positive gene targets. As for ChIP-seq and Hi-C libraries, size distribution is checked on agarose gel and Fragment Analyzer (FA). During data analysis, we calculate the correlation among replicates and compare IGV track view of replicates. All these measures approve the stability of replicates.
Randomization	By Randomization
Blinding	We were blinded to group allocation

Reporting for specific materials, systems and methods

We require information from authors about some types of materials, experimental systems and methods used in many studies. Here, indicate whether each material, system or method listed is relevant to your study. If you are not sure if a list item applies to your research, read the appropriate section before selecting a response.

Materials & experimental systems

n/a	Involved in the study	
[ ]	[x]	Antibodies
[x]	[ ]	Eukaryotic cell lines
[x]	[ ]	Palaeontology and archaeology
[ ]	[x]	Animals and other organisms
[x]	[ ]	Human research participants
[x]	[ ]	Clinical data
[x]	[ ]	Dual use research of concern

Methods

n/a	Involved in the study	
[ ]	[x]	ChIP-seq
[ ]	[x]	Flow cytometry
[x]	[ ]	MRI-based neuroimaging

Antibodies

Antibodies used	H3K4me3: Millipore, Cat.No: 04-745, Lot.No: 2872328.<br>H3K4me1: Abcam, Cat.No: ab8895, Lot.No: GR3206758-1.<br>H3K27me3: Millipore, Cat.No: 07-449, Lot.No: 2194165.<br>H3K27ac: Diagenode, Cat.No: c15410196, Lot.No: A1723-0041D.<br>RUNX1: Abcam, Cat.No: ab23980, Lot.No: GR3213439-1.
Validation	All primary antibodies are validated by the manufacturer's website as shown above.<br>We validated antibodies by large and low number cell itChIP in mESCs or sorted mouse in vivo hematopoietic cells.

Animals and other organisms

Policy information about studies involving animals; ARRIVE guidelines recommended for reporting animal research

Laboratory animals	Species: mouse. Strain:C57BL/6. Sex: male and female. Ages: 2-3 month old.<br>Mouse embryos at different stages were identified by somite numbers and collected.
Wild animals	The study did not involve wild animals.
Field-collected samples	The study did not involve samples collected from the field.
Ethics oversight	The Institutional Animal Care and Use Committee of Peking University and Academy of Military Sciences.

Note that full information on the approval of the study protocol must also be provided in the manuscript.

ChIP-seq

Data deposition

- [x] Confirm that both raw and final processed data have been deposited in a public database such as GEO.
- [x] Confirm that you have deposited or provided access to graph files (e.g. BED files) for the called peaks.

Data access links<br>*May remain private before publication.*	Sequencing data have been deposited at the NCBI Gene Expression Omnibus (GEO) under accession GSE161328, at https://www.ncbi.nlm.nih.gov/geo/query/acc.cgi?acc=GSE161328
Files in database submission	AEC-H3K27ac_rep1_R1.fq.gz<br>AEC-H3K27ac_rep1_R2.fq.gz<br>AEC-H3K27ac_rep2_R1.fq.gz<br>AEC-H3K27ac_rep2_R2.fq.gz<br>AEC-H3K27me3_rep1_R1.fq.gz<br>AEC-H3K27me3_rep1_R2.fq.gz<br>AEC-H3K27me3_rep2_R1.fq.gz<br>AEC-H3K27me3_rep2_R2.fq.gz<br>AEC-H3K4me1_rep1_R1.fq.gz<br>AEC-H3K4me1_rep1_R2.fq.gz<br>AEC-H3K4me1_rep2_R1.fq.gz<br>AEC-H3K4me1_rep2_R2.fq.gz<br>AEC-H3K4me3_rep1_R1.fq.gz<br>AEC-H3K4me3_rep1_R2.fq.gz<br>AEC-H3K4me3_rep2_R1.fq.gz<br>AEC-H3K4me3_rep2_R2.fq.gz<br>AEC-RUNX1_rep1_R1.fq.gz<br>AEC-RUNX1_rep1_R2.fq.gz<br>AEC-RUNX1_rep2_R1.fq.gz<br>AEC-RUNX1_rep2_R2.fq.gz<br>AEC-RUNX1_rep3_R1.fq.gz<br>AEC-RUNX1_rep3_R2.fq.gz<br>HEC-H3K27ac_rep1_R1.fq.gz<br>HEC-H3K27ac_rep1_R2.fq.gz<br>HEC-H3K27ac_rep2_R1.fq.gz<br>HEC-H3K27ac_rep2_R2.fq.gz<br>HEC-H3K27me3_rep1_R1.fq.gz<br>HEC-H3K27me3_rep1_R2.fq.gz<br>HEC-H3K27me3_rep2_R1.fq.gz<br>HEC-H3K27me3_rep2_R2.fq.gz<br>HEC-H3K4me1_rep1_R1.fq.gz<br>HEC-H3K4me1_rep1_R2.fq.gz<br>HEC-H3K4me1_rep2_R1.fq.gz<br>HEC-H3K4me1_rep2_R2.fq.gz<br>HEC-H3K4me3_rep1_R1.fq.gz<br>HEC-H3K4me3_rep1_R2.fq.gz<br>HEC-H3K4me3_rep2_R1.fq.gz<br>HEC-H3K4me3_rep2_R2.fq.gz<br>HEC-RUNX1_rep1_R1.fq.gz<br>HEC-RUNX1_rep1_R2.fq.gz<br>HEC-RUNX1_rep2_R1.fq.gz<br>HEC-RUNX1_rep2_R2.fq.gz<br>preHSC-H3K27ac_rep1_R1.fq.gz

preHSC-H3K27ac_rep1_R2.fq.gz
preHSC-H3K27ac_rep2_R1.fq.gz
preHSC-H3K27ac_rep2_R2.fq.gz
preHSC-H3K27me3_rep1_R1.fq.gz
preHSC-H3K27me3_rep1_R2.fq.gz
preHSC-H3K27me3_rep2_R1.fq.gz
preHSC-H3K27me3_rep2_R2.fq.gz
preHSC-H3K4me1_rep1_R1.fq.gz
preHSC-H3K4me1_rep1_R2.fq.gz
preHSC-H3K4me1_rep2_R1.fq.gz
preHSC-H3K4me1_rep2_R2.fq.gz
preHSC-H3K4me3_rep1_R1.fq.gz
preHSC-H3K4me3_rep1_R2.fq.gz
preHSC-H3K4me3_rep2_R1.fq.gz
preHSC-H3K4me3_rep2_R2.fq.gz
preHSC-RUNX1_rep1_R1.fq.gz
preHSC-RUNX1_rep1_R2.fq.gz
preHSC-RUNX1_rep2_R1.fq.gz
preHSC-RUNX1_rep2_R2.fq.gz
LTHSC-H3K27ac_rep1_R1.fq.gz
LTHSC-H3K27ac_rep1_R2.fq.gz
LTHSC-H3K27ac_rep2_R1.fq.gz
LTHSC-H3K27ac_rep2_R2.fq.gz
LTHSC-H3K27ac_rep3_R1.fq.gz
LTHSC-H3K27ac_rep3_R2.fq.gz
LTHSC-H3K27me3_rep1_R1.fq.gz
LTHSC-H3K27me3_rep1_R2.fq.gz
LTHSC-H3K27me3_rep2_R1.fq.gz
LTHSC-H3K27me3_rep2_R2.fq.gz
LTHSC-H3K4me1_rep1_R1.fq.gz
LTHSC-H3K4me1_rep1_R2.fq.gz
LTHSC-H3K4me1_rep2_R1.fq.gz
LTHSC-H3K4me1_rep2_R2.fq.gz
LTHSC-H3K4me3_rep1_R1.fq.gz
LTHSC-H3K4me3_rep1_R2.fq.gz
LTHSC-H3K4me3_rep2_R1.fq.gz
LTHSC-H3K4me3_rep2_R2.fq.gz
LTHSC-H3K4me3_rep3_R1.fq.gz
LTHSC-H3K4me3_rep3_R2.fq.gz
LTHSC-RUNX1_rep1_R1.fq.gz
LTHSC-RUNX1_rep1_R2.fq.gz
LTHSC-RUNX1_rep2_R1.fq.gz
LTHSC-RUNX1_rep2_R2.fq.gz

Genome browser session
(e.g. UCSC)

no longer applicable

Methodology

Replicates

Two or three biological replicates for ChIP-seq libraries.

Sequencing depth

All the libraries were sequenced by PE150 for 3-6 G raw reads.

Antibodies

H3K4me3: Millipore, Cat.No: 04-745, Lot.No: 2872328.
H3K4me1: Abcam, Cat.No: ab8895, Lot.No: GR3206758-1.
H3K27me3: Millipore, Cat.No: 07-449, Lot.No: 2194165.
H3K27ac: Diagenode, Cat.No: c15410196, Lot.No: A1723-0041D.
RUNX1: Abcam, Cat.No: ab23980, Lot.No: GR3213439-1.

Peak calling parameters

Peaks were identified using MACS2 with the parameter "--broad --nomodel --nolambda".

Data quality

We evaluated the data quality by track view, calculating the correlation of different groups and etc.

Software

FastQC (v 0.11.5), Cutadapt (v 1.11), Bowtie2 (v 2.2.9), Deeptools (v 2.2.3). Samtools (v 1.9), Picard (v.2.2.4), MACS2 (v.2.2.5)

Flow Cytometry

Plots

Confirm that:

- [x] The axis labels state the marker and fluorochrome used (e.g. CD4-FITC).
- [x] The axis scales are clearly visible. Include numbers along axes only for bottom left plot of group (a 'group' is an analysis of identical markers).
- [x] All plots are contour plots with outliers or pseudocolor plots.
- [x] A numerical value for number of cells or percentage (with statistics) is provided.

Methodology

Sample preparation	Trypsin was used for tissue dissociation at 37°C. The dissociated cells were incubated with antibody mixture at 4°C for 30 min, followed by incubation with 7-AAD antibody at room temperature for 5 min.
Instrument	Cells were sorted and analyzed by flow cytometers FACS Aria II (BD Biosciences) and MoFlo XDP (Beckman Coulter) in the purity model.
Software	The FACS data were analyzed with FlowJo software (V10, Tree star).
Cell population abundance	Surface markers for E10.0 early AECs in AGM regions were CD41-CD43-CD45-CD31+CD44+Kit-.<br>Surface markers for E10.0 HECs in AGM regions were CD41-CD43-CD45-CD31+CD44+Kit+CD201+.<br>Surface markers of E11.0 AGM pre-HSCs were CD31+Kit+CD201+.<br>Surface markers for E14.5 fetal liver LT-HSCs were CD45+CD201+CD150+CD48-.
Gating strategy	The compensation was adjusted for between channels. FSC and SSC were used to selected single and alive cells. As for surface markers, cells can be obviously separated into split patterns indicating positive and negative populations.

- [x] Tick this box to confirm that a figure exemplifying the gating strategy is provided in the Supplementary Information.
